# PReS-FINAL-2105: Can routine clinical and laboratory data predict JIA diagnosis and disease course in acute arthritis?

**DOI:** 10.1186/1546-0096-11-S2-P117

**Published:** 2013-12-05

**Authors:** S Rusoniene, V Panaviene

**Affiliations:** 1 Pediatric, Children Hospital, Affiliate of Vilnius University Hospital Santariskiu Klinikos, Vilnius, Lithuania

## Introduction

The assessment of a child with acute join pain and swelling needs to differentiate between various conditions. JIA is the most common form of childhood arthritis and one of the most common chronic childhood illnesses. JIA subtypes demonstrate unique clinical presentations and clinical courses. The pain and functional disability associated with JIA cause significant burden for patients and their families, affecting on health-related quality of life and well-being. Therefore, JIA should be recognised and treatment started as soon as possible. Early diagnosis is a determinant step in the management of these patients to prevent the effects of an illness.

## Objectives

To investigate clinical and laboratory manifestations of acute early arthritis and determine early predictors of JIA

## Methods

Early arthritis programme is performed in the Vilnius University Children Hospital, Pediatric Rheumatology Department. Patients from whole Lithuania with acute arthritis (disease's duration from 1 weeks to 3 months) symptoms are included. Special questionnaire, CHAQ, general status, active joint count, systemic manifestations, immunological parameters (ANA, HLA B27, RF), acute phase reactants, serology (*Yersinia, Salmonella, ASL*) are assessed at the first visit and after 6, 12, 24 months of follow up.

## Results

106 patients (from 2010-2013 years) with acute arthritis symptoms were enrolled in the study, 60 girls and 46 boys. Range of age was from 1 to 17 years (mean 8, 32 + 5,053 years) with the median disease period from the first symptoms 6,2 + 3,45 weeks. Monoarthritis was observed in 55, 9% patients, 29% had oligoarthritis-onset and 15, 1% polyarthritis-onset disease course. Morning stiffness was absent in 25% of children. 25 patients (23, 4%) were ANA positive at high titre (19/25 - 1:200). ANA were most prevalent in polyarthritis subgroup (27, 5%) and predominantly with a diffuse granular pattern. Also ANA was more common for boys more then 9 years. HLA B27 positive patients were 23, 4% (28% had both ANA and HLA B27, equally in subgroups). The diagnosis was determined according to the International League of Associations for Rheumatology (ILAR) 1997 classification during follow up period after 6 month from disease onset. Juvenile idiopathic arthritis was the most frequent diagnosis (50%), undifferentiated arthritis - 25, 5%, reactive cause of arthritis - 20, 75%. Systemic lupus erythematosus, leukemia, sarcoma were single cases. At the time of first symptoms pain severity was similar among all types of arthritis. Children with JIA reporter less pain and greater wellbeing than other children with others diseases, however rated more disability of quality of life (CHAQ median 0, 785 + 0, 75).

## Conclusion

In our cohort clinical and routine laboratory (also ANA, HLA B27) parameters couldn't early predict particular JIA form. It may be relationship between acute arthritis symptoms and quality of life. Also ANA and HLA B27 were not so frequent and predominated more in older boys. The study is ongoing and further more sensitive and informative early disease predictors should be investigated.

## Disclosure of interest

None declared.

